# Novel Biobased Copolymers Based on Poly(butylene succinate) and Cutin: In Situ Synthesis and Structure Properties Investigations

**DOI:** 10.3390/polym16162270

**Published:** 2024-08-10

**Authors:** Evangelia D. Balla, Panagiotis A. Klonos, Apostolos Kyritsis, Monica Bertoldo, Nathanael Guigo, Dimitrios N. Bikiaris

**Affiliations:** 1 Laboratory of Polymer Chemistry and Technology, Department of Chemistry, Aristotle University of Thessaloniki, 54124 Thessaloniki, Greece; pklonos@chem.auth.gr; 2 Department of Physics, National Technical University of Athens, Zografou Campus, 15780 Athens, Greece; akyrits@central.ntua.gr; 3 Department of Chemical, Pharmaceutical and Agricultural Sciences, University of Ferrara, 44121 Ferrara, Italy; brtmnc@unife.it; 4 Institute of Chemistry, Université Côte d’Azur, UMR 7272, 06108 Nice, France; nathanael.guigo@univ-cotedazur.fr

**Keywords:** poly(butylene succinate), cutin, copolyesters, synthesis, biodegradation

## Abstract

The present work describes the synthesis of poly(butylene succinate) (PBSu)-cutin copolymers by the two-stage melt polycondensation method, esterification and polycondensation. Cutin was added in four different concentrations, 2.5, 5, 10, and 20 wt%, in respect to succinic acid. The obtained copolymers were studied using a variety of techniques such as Fourier transform infrared spectroscopy (FTIR), X-ray diffraction analysis (XRD), differential scanning calorimetry (DSC), thermogravimetric analysis (TGA), polarized light microscopy (PLM), as well as diffuse reflectance spectroscopy (DRS). A series of results, in agreement between different techniques, revealed the formation of PBSu-cutin interactions, confirming indirectly the successful in situ synthetic route of copolymers. DSC and XRD combined with PLM results provided indications that the crystallization temperature increases with the addition of small amounts of cutin and gradually decreases with increasing concentration. The crystallization process was easier and faster at 2.5%, 5%, and 10% concentrations, whereas at 20%, it was comparable to neat PBSu. The presence of cutin, in general, leads to the facilitated crystallizability of PBSu (direct effect), whereas a moderate drop in the glass transition temperature is recorded, the latter being an indirect effect of cutin via crystallization. The thermal stability improved in the copolymers compared to neat PBSu. Water contact angle measurements confirmed that the addition of cutin decreased the hydrophilicity. The local and segmental relaxation mapping is demonstrated for PBSu/cutin here for the first time. Enzymatic hydrolysis and soil degradation tests showed that, overall, cutin accelerated the decomposition of the polymers. The copolymers may be proven useful in several applications.

## 1. Introduction

The synthesis of aliphatic polyesters remains an aspect of considerable interest in various fields, particularly in biomedicine, tissue engineering, food packaging, agriculture, etc. They usually find applications in drug delivery systems, wound closure products, surgical implants, and other areas such as food containers, agricultural films, and waste bags. However, many of these polyesters exhibit characteristics such as crystallinity, hydrophobicity, and lack of free functional groups, which limits their versatility for further modification. One possible approach in overcoming these limitations involves introducing new functional groups into the polymer structure. Incorporating free functional groups, e.g., hydroxyls and carboxylates, can significantly enhance the hydrophilicity and physical degradability of polyesters, while also allowing adjustments in their mechanical, thermal, chemical, and biological properties. Moreover, these functional groups offer the opportunity for attaching various bioactive molecules, thus creating novel biomaterials with a wide range of potential applications. Binding may be enhanced by adding hydroxyl- or carboxyl-functional pendant groups to the macromolecular chains of these polymers. For example, trifunctional alcohols and divinyl acid derivatives were used to create hydroxyl-functionalized materials by Kline et al. [1] and Uyama et al. [2]. Furthermore, Kumar et al. [3] demonstrated the direct condensation of a diacid with glycerol or sorbitol to produce materials that have been functionalized with hydroxyls. The majority of those materials were created by polymerizing lactides and lactones with ring opening, as well as compounds including hydroxyl, carboxyl, amino, and cyclic esters and anhydrites. Even though useful, their non-linear structure places restrictions on their solubility, crystallinity, polarity, and reactivity.

Research on aliphatic polyesters utilizing renewable and biodegradable resources has been prompted by the demand for a wider range of characteristics. Biopolymers are an interesting substitute for typical non-degradable polymers since they are biodegradable while their primary sources are renewable, like agro-resources. In this context, biomass is an abundant renewable resource that is carbon neutral and may be used to produce biomaterials. Under proper circumstances, many of these polymers derived from renewable resources can be made biodegradable [4]. Lately, researchers focus on biodegradable polymers in various fields. In medicine, bioplastics are used by exploiting their ability to break down gradually in the body without leaving behind any foreign matter. Among them, aliphatic polyesters including poly(lactic acid) (PLA), poly(glycolic acid) (PGA), poly(ε-caprolactone) (PCL), and poly(p-dioxanone) (PPDO) are widely used in biomedical disciplines and clinical practice. Another aliphatic polyester, which has gained attention recently due to potential medical applications, is poly(butylene succinate) (PBSu). PBSu is a biodegradable thermoplastic polyester that is easily synthesized when 1,4 butanediol and succinic acid condense [5]. Because its two components may be produced by biologically fermenting sugar cane, cassava, and maize, it is also a sustainable and biobased polyester. 

PBSu is widely used in industry and daily life due to its good mechanical properties, thermostability, and thermal processing ability. It has also been at the epicenter of numerous medical application studies, including those on drug delivery, bone repair, cartilage tissue engineering, cardiac tissue engineering, and antibacterial function [6]. PBSu performs with outstanding biodegradation characteristics because of its easily hydrolyzable ester groups. Moreover, it can be hydrolyzed by a wide range of microbes and enzymes produced in plants and animals under particular microbial exposure conditions, such as composting, which results in the production of water and carbon dioxide while preventing environmental contamination. Biodegradation can be affected by the enzymes’ type, the polyester’s molecular weight, and the degree of crystallinity. PBSu performs well overall, but its drawbacks include low stiffness and high production cost. PBSu must be altered in order to improve its function, lower its production costs, and enable PBSu to be used broadly [7]. Herein, due to its structural features, a cutin monomer is studied as a promising agro-waste bioproduct for the production of PBSu-based copolymers [7].

The amount of agricultural waste from harvestable areas is a major issue since food loss and waste have the greatest environmental impact associated with this business. Cutin is a biopolymer in abundance formed from plants that is found in plant cuticles, such as tomato skins, from which many monomers have been identified and extracted [8,9]. Cutin, the most common polyester discovered in nature, forms the framework of the protective cuticle that protects the stems and leaves of higher plants [10,11,12]. The primary constituents of this non-toxic, biodegradable, crosslinked polyester are polyhydroxylated C16 and C18 fatty acids. The analysis of specific monomers in polymerization reactions has been claimed impossible due to the complexity and variances between different species despite various attempts to extract the monomers which are present in cutin [8], but there are not many published studies on polymerization processes with cutin monomers derived from tomato treatment [13]. Considering its properties (non-toxic, biobased, biocompatible, antimicrobial [14]), several research works aimed to develop new plant cuticle-inspired polyesters, such as coating layers for food cans and hydrophobic films for packaging applications, as well as to produce cutin oligomers with enhanced antimicrobial properties. On the other hand, owing to its structural features (hydroxyl- and carboxyl-free functional pendant), cutin monomers could also be promising for the production of PBSu-based copolymers [15].

In the present study, the topic of investigation originates from the increasing interest in renewable polymers and the valorization of feedstock byproducts as well as our objective of enhancing their properties. As a result, a series of new copolyesters based on PBSu and cutin, in different ratios (2, 5, 10, and 20%), was synthesized via a two-stage melt polycondensation. The writers would like to clarify that although the present study focuses on PBSu synthesis from feedstock byproducts, the reagents used (succinic acid and 1,4-butanediol) were of analytical grade to ensure accurate and reproducible results. This approach can establish the data for applying the findings and understanding in biobased materials. For the investigation of the structure and properties of PBSu/cutin-obtained polyesters, a variety of techniques were employed, including Fourier transform infrared spectroscopy (FTIR), X-ray diffraction (XRD), differential scanning calorimetry (DSC), dielectric relaxation spectroscopy (DRS), thermogravimetric analysis (TGA), water contact angle, and polarized light microscopy (PLM). Additionally, their degradation rate during soil degradation and enzymatic hydrolysis was also studied. To the best of our knowledge, no scientific studies of PBSu and cutin based polymers have yet been examined by other scientific groups according to the literature. 

## 2. Materials and Methods

### 2.1. Materials

Succinic acid (SA) (purity: ≥99.5%), and titanium isopropoxide (purity 97%) (Tis) catalyst of an analytical grade were obtained from Sigma–Aldrich Chemical Co. (Saint Louis, MO, USA). 1,4-Butanediol (BD) (Purity: >99%) and were purchased from Thermo Scientific (Waltham, MA, USA). The cutin used in this work was kindly donated by the company Tomapaint, Parma, Italy. Cutin was freeze-dried prior to use in order to avoid excess of water in the reaction mixture. All other reagents were of analytical grade.

### 2.2. Synthesis

#### 2.2.1. Synthesis of Poly(butylene succinate)

**Figure 1:**
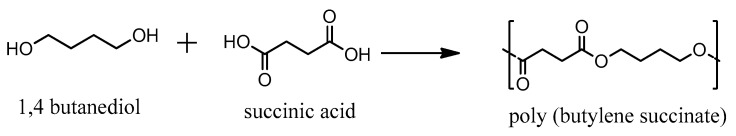
Synthesis of PBSu using succinic acid and 1.4-butanediol.

The synthesis of PBSu was carried out by the two-stage melt polycondensation method, containing an esterification and a polycondensation (Figure 1). The melt polycondensation method was employed aiming to facilitate the byproducts removal and to achieve control over reaction conditions to finally obtain the polymer with the desired properties. SA and BD were used in a molar ratio of 1.2/1, and were charged into a 250 mL glass batch reactor. Vacuum and nitrogen were applied three times in the reaction mixture in order to obtain an idle environment. The polymerization mixture was heated at 170 °C under nitrogen for 1 h and was gradually increased to 220 °C every hour. The stirring speed was set at 350 rpm. The esterification step was completed when all of the theoretical amount of H_2_O had been distilled. In the second reaction step, 400 ppm of Tis catalyst was added to the reactor and vacuum was increased to 5.0 Pa slowly, in order to avoid excessive foaming, aiming to remove the excess diol or remaining H_2_O, and minimize oligomer sublimation. During this polycondensation step, the temperature was gradually increased up to 230 °C, while the stirring speed was also increased to 520 rpm. The reaction was maintained for 30 min at this temperature, which was then increased to 240 °C, where the reaction was carried out for another hour.

#### 2.2.2. Synthesis of PBSu/Cutin Copolymers

**Figure 2:**
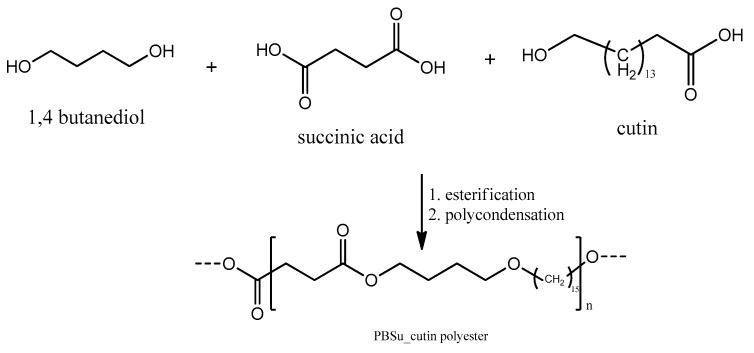
Synthesis of PBSu/cutin polyesters using succinic acid and 1.4-butanediol and cutin.

**Figure 3:**
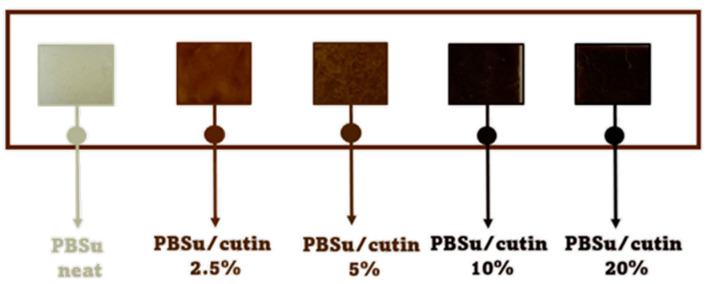
Gradual coloration of the obtained products due to cutin.

In a similar way to the procedure described above, PBSu/cutin copolymers were produced by in situ polymerization containing 2.5, 5, 10, and 20 wt% cutin (Figure 2) [16]. Cutin was added in the first step of the polymerization along with SA and BD. The ratios of materials used are described in Table 1. Owing to cutin, the obtained materials exhibit a characteristic dark brown color (Figure 3).

#### 2.2.3. Fourier Transform Infrared Spectroscopy (FTIR)

The chemical structure of the PBSu/cutin copolymers was determined with FTIR spectroscopy. FTIR spectroscopy was carried out on a Cary 670 spectroscope from Agilent Technologies (Palo Alto, CA, USA) equipped with a diamond attenuated total reflectance (ATR) accessory, (GladiATR, Pike Technologies, Madison, WI, USA). The spectra were collected in the area 4000–400 cm^−1^, with 16 scans and a resolution of 4 cm^−1^.

#### 2.2.4. X-ray Powder Diffraction (XRD)

For the investigation of any effects on the structure, measurements by XRD at room conditions were carried out over the 2θ range from 5° to 45° at the scanning rate of 1°/min in a MiniFlex II XRD-diffractometer (Chalgrove, Oxford, UK), with Cu Ka radiation for crystalline phase identification (λ = 0.154 nm). 

#### 2.2.5. Differential Scanning Calorimetry (DSC)

Conventional DSC measurements were performed in a high purity nitrogen atmosphere, within the temperature range from −110 to 140 °C by means of a TA Q200 calorimeter (TA Instruments, New Castle, DE, USA), being previously calibrated with sapphires for heat capacity and indium for temperature and enthalpy. The samples, ~8 mg in mass, were closed in Tzero aluminium pans using TA. Upon the erasing of samples’ thermal history by heating at *T* = 160 °C (i.e., at *T* = *T*_m_ + 50 °C), the samples were subjected to cooling at 20 °C/min and a subsequent heating at 10 °C/min.

#### 2.2.6. Dielectric Relaxation Spectroscopy (DRS)

The DRS [17] measurements were conducted in gas nitrogen atmosphere by means of a Novocontrol (Novocontrol GmbH, Montabaur Germany) DRS setup, namely, an Alpha Analyzer combined with a Quatro liquid nitrogen cryosystem (temperature control better than 0.5 K) on samples of ~16 mm in diameter and ~1.5 mm in thickness. The polymer-based samples were placed between finely polished brass electrodes (disks) forming a sandwich-like capacitor. The dielectric/electrical response was recorded using the WinDETA software of Novocontrol, in particular, the complex dielectric permittivity, ε* = *ε*′ − i·*ε*′′, as a function of frequency in the range from 10^−1^ to 10^6^ Hz and in the temperature range between −140 and 40 °C upon heating at steps of 5 and 10 K.

#### 2.2.7. Thermogravimetric Analysis (TGA)

Thermogravimetric analysis (TGA) measurements were performed on the TGA2 from Mettler Toledo, Colombus, USA. A precision of ±0.1 µg is obtained from the microbalance. Samples subject to thermal stability analysis were weighed between 10 and 20 mg and placed into 70 μL alumina pans. A thermal program going from 0 °C to 650 °C was applied on the samples with a heating rate of 20 °C/min. All the experiments were conducted under air flow (50 mL/min).

#### 2.2.8. Water Contact Angle Measurements

In order to measure the surface wettability of the copolymers, measurements were carried out using the Ossila Contact Angle Goniometer L2004A1 water contact angle tester. The sessile droplet method was used to analyze the hydrophilicity of the prepared materials, i.e., 25 μL of distilled water was added dropwise onto the top surface of the samples. Thin films of the materials were prepared in order to receive an even surface. The measurements were performed by video recording and measuring the angle formed by the drop with the surface of the material at time t = 0 s and t = 10 s with a high-resolution camera, and were processed using the Ossila Contact Angle Software 4.1.4 (Ossila Ltd., Sheffield, UK). At least three measurements were conducted, and the mean value is presented.

#### 2.2.9. Polarized Light Microscopy (PLM)

PLM was employed to provide a view of the surface morphology, namely, the distribution and sizes of the formed crystals during cold crystallization. The materials underwent initial heating just above their melting temperature (*T = T_m_ + 20 °C*) to ensure uniform melting. Subsequently, the materials were cooled, at 10 °C/min, imitating, thus, the thermal protocols employed in DSC for scans. PLM measurements were conducted during isothermal annealing to observe variations in the size and population of the resulting spherulites. The PLM images were obtained using a Nikon Optiphot-2 polarizing light microscope which was equipped with a Linkam THMS 600 heating stage, a Linkam TP 91 control unit, and a Jenoptic ProgRes C10Plus capturing camera with the Jenoptik ProgRes CapturePro C10Plus software.

#### 2.2.10. Scanning Electron Microscopy (SEM)

SEM studies of the prepared materials were carried out using a JEOL JMS 7610F (Freising, Germany) scanning microscope equipped with an energy dispersive X-ray (EDX) Oxford ISIS 300 micro-analytical system. SEM pictures were captured before and after enzymatic hydrolysis and soil degradation of the prepared samples in order to observe their behavior during decomposition. 

#### 2.2.11. Enzymatic Hydrolysis

The enzymatic hydrolysis of the prepared copolymers took place in aqueous solution of Rhizopus Arrhizus and Pseudomonas Cepacia lipases. For this purpose, the enzymatic hydrolysis of the prepared copolymers was determined by measuring the mass loss of specimens of similar size and weight, in 10 mL of PBS buffer containing the aforementioned enzymes placed in tubes and kept at 37 °C. The specimens were taken out every 10 days, washed with water and dried under vacuum at 50 °C to a constant mass. Each measurement was repeated 3 times. The enzymatic hydrolysis degree of the copolymers was estimated by the average sample weight loss. 

#### 2.2.12. Soil Barrier

The biodegradability of the prepared copolymers in soil was determined by measuring the mass loss of specimens buried in conventional leaf mold of the same size and similar thickness. Each specimen was buried in soil at room temperature (about 30 °C). Water was supplied regularly so that the soil was kept moisturized. Each specimen was taken out of the soil every 10 days. Obtained films were washed with distilled water and dried at 60 °C under vacuum until constant mass was reached. The degree of soil degradation was estimated by the average sample weight loss as the mean value of 3 measurements.

## 3. Results and Discussion

### 3.1. Fourier Transform Infrared Spectroscopy (FTIR)

All copolymers have been extensively characterized using several techniques. Unfortunately, it was not possible to measure the molecular weight of copolymers since all are insoluble in any solvent. This is an indication that probably some crosslinked or highly branched materials have been produced during copolymer synthesis. This is because, as reported in the literature, cutin consists of many monomers, mostly of difunctional hydroxy fatty acids, a,ω-diacids, and hydroxy-epoxy fatty acids, and multifunctional glycerol (up to 14% of monomers), di- or three-hydroxy acids, and hydroxy-epoxy-acids [18,19,20,21,22]. All these monomers have multifunctional groups that can lead to branched or crosslinked materials. For this reason, the chemical structure of prepared copolymers was not characterized by NMR but only by FTIR.

**Figure 4:**
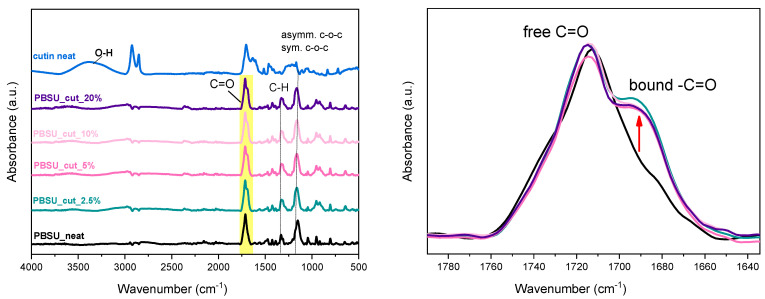
(**Left**) ATR-FTIR spectroscopy, (**right**) details on the ester group vibration peak.

In Figure 4, the FTIR spectra for all samples are comparatively presented, including neat cutin and PBSu. The characteristic PBSu-originating bonds are present in PBSu/cutin polyesters as recorded by the FTIR peaks at 1710, 1470, 1410, 1330, 1150 cm^−1^. Cutin shows, among others, a characteristic broad peak at around 3300 cm^−1^ corresponding to its various –OH groups (free, bound, arising from cutin, interfacial water molecules, etc.). Two main contributions in the carbonyl adsorption band at around 1700 cm^−1^, can be detected, the most intense of which is assigned to carboxylic acid groups [23]. The shoulder at higher frequency can be assigned to oligoesters. The peaks attributed to the OH of cutin moieties (OH-(CH_2_)_15_COOH) are not observed in the final materials, as described from the reaction in Figure 2. The contribution of cutin can be traced by the peak at 1700 cm^−1^ corresponding to the ester group C=O, which is the most polar site of both polymers. Interestingly, in the right Figure 4, there seems to be an increasing contribution of the ester vibrations at lower wavenumbers in PBSu/cutin, as compared to neat PBSu and initial cutin. According to similar observations in the past for different polyesters [24,25,26], this effect has been attributed to the increasing fraction of ‘bound’ ester groups. In our case, this could suggest an increased level of interaction between the two polymers. This is actually expected here and provides strong evidence of the successful in situ polymerization (i.e., PBSu chains being developed over cutin sites).

### 3.2. X-ray Powder Diffraction (XRD)

**Figure 5:**
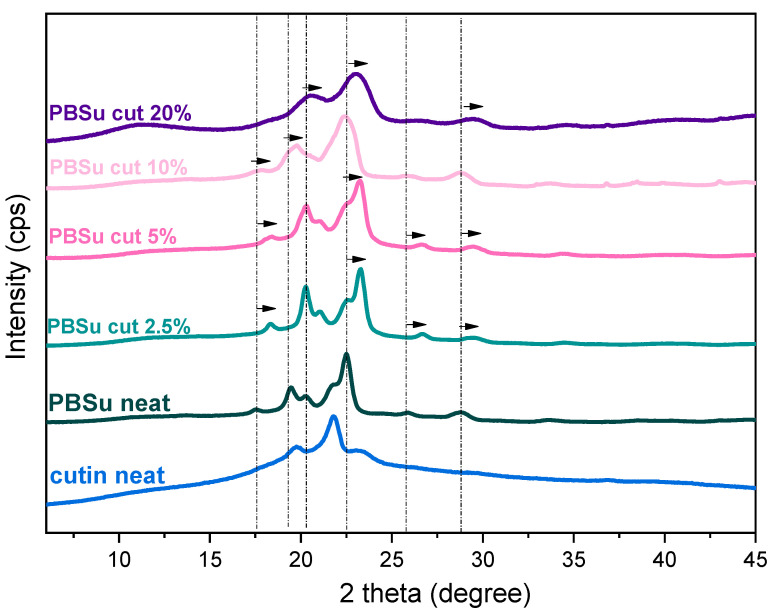
XRD profiles of PBSu/cutin copolyesters.

In Figure 5, we present the overall XRD spectra. Both PBSu neat and cutin neat are semi-crystalline. Please note that PBSu is a strongly crystallizable polymer [27,28]. The corresponding main X-ray diffraction peaks for PBSu and cutin are recorded at 20.1, 21.3, and 23.2°, and at 19.7, 21.7, and 23.1°, respectively [29]. These peaks are also observed at PBSu/cutin 2.5 and 5 wt%. The results from materials 10 and more at 20 wt% show a wider curve. Their peaks are shifted to 20 and 22 degrees. Taking a more careful look at Figure 5, we may conclude in the PBSu/cutin copolymers the main diffraction peaks are slightly migrated toward higher 2*θ* positions, as compared to initial PBSu, which suggests the formation of smaller unit cells [30,31]. Such effect in polymers has been explained, in combination with other techniques (calorimetry, microscopy) to the formation of less dense or/and smaller crystal spherulites [25,27]. Such seems to be the case here as will be seen in the following, by DSC and PLM results. In particular, the result is consistent with DSC measurements according to which, at low concentrations of cutin, a shoulder peak is observed, and their second melting peak no longer exists. XRD measurements for PBSu/cutin 10 and 20 wt% revealed their semicrystalline character, which is supportive to the strong crystallizability of PBSu, despite the copolymerization and the chain length alternations.

### 3.3. Differential Scanning Calorimetry (DSC)

**Figure 6:**
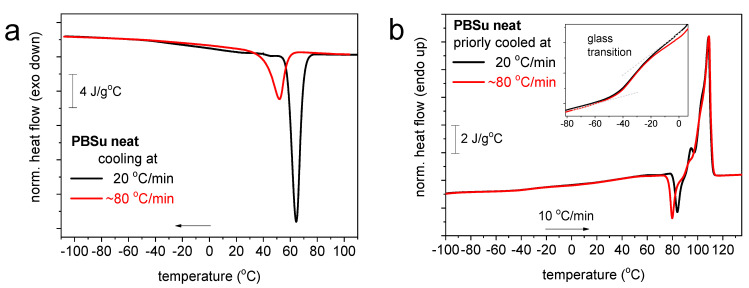
DSC (**a**) cooling and subsequent (**b**) heating traces for PBSu and the conditions described within the figures.

**Figure 7:**
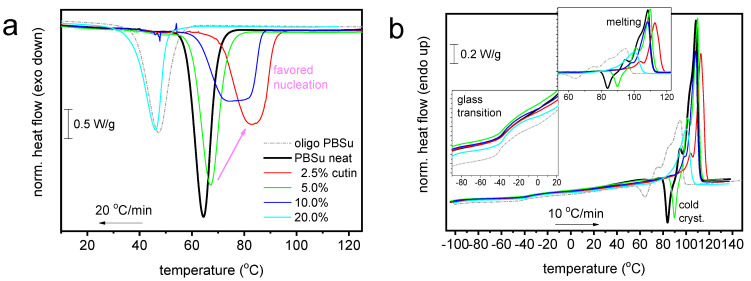
Comparative DSC traces for all PBSu-based systems during (**a**) cooling at 20 °C/min and (**b**) subsequent cooling at 10 °C/min. The insets to (**b**) provide details of temperature regions of glass transition and melting. For comparison, included are the results.

In Figure 6 and Figure 7 we present the results by differential scanning calorimetry for neat PBSu and PBSu/cutin copolymers, respectively. Due to the semicrystalline nature of PBSu and in order to extract as direct as possible effects by the presence of cutin, we attempted to vanish crystallization. This was attempted by a fast cooling in conventional DSC (Figure 6a). Crystallization could be suppressed but not eliminated completely. This was more or less expected for PBSu [27,28]. Despite the suppression of melt-crystallization, the subsequent cold-crystallization upon heating was enhanced. Independently from the slower and the fast cooling, the glass transition temperature, *T*_g_ = −33 °C, and melting temperature, *T*_m_ = 108 °C, were practically unchanged.

Coming to the effects on the thermal transitions imposed by the presence of cutin, we turn the attention to Figure 7. Therein, all the thermal transitions of PBSu could still be recorded in the copolymers, nevertheless exhibiting worth-noting alternations. The thermal events were evaluated and the basic values are listed in Table 2.

In Figure 7a, melt crystallization is mainly accelerated in the copolymers (elevation of *T*_c_), with the exception of 20 wt% cutin. The effect suggests facilitated crystal nucleation, which is optimized for 2.5 wt% cutin. In some cases of PBSu/cutin, there seems to be a double-shaped crystallization (superposition of two peaks). The low temperature peak view resembles actually that of the oligomeric butylene succinate. This could be related with the possibility of remaining oligomers next to PBSu in said copolymer. Despite that, the estimated crystalline fraction (CF_c_) is mainly constant (~37–39%) with the exception of PBSu/cutin 20 wt% (CF_c_~30%). The increasing *T*_c_ combined with unchanged CF_c_ suggests the alternation in the semicrystalline morphology; in particular, in the copolymers, we mainly expect more crystals of smaller dimensions as compared to initial PBSu. Partly, this is confirmed in a following section by PLM.

Coming to the polymer’s mobility, we focus now on the glass transition. The characteristic temperature, *T*_g_, of PBSu (−33 °C) exhibits mainly a mild drop in the copolymer. Obviously, this is not expected as a direct effect of cutin on PBSu. It is, most probably, an indirect effect arising from the superposition of effects due to changes in crystallinity and/or semicrystalline morphology accompanied by the lowering of the chain lengths. The latter effect is more pronounced in the extreme case of PBSu/cutin 20 wt%.

A variety of effects are also recorded on melting. Melting is recorded in general as a complex endothermal peak (double-triple shaped) in all cases. The maximum melting temperature, *T*_m,max_, of PBSu is moderately increased for low cutin ratios and tends to decrease for the higher ones. Again, as discussed within crystallization and glass transition, more than one parameter co-affects melting. 

### 3.4. Molecular Mobility (DRS) 

**Figure 8:**
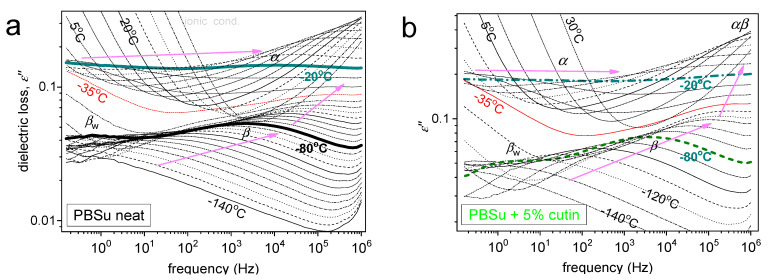
DRS results of ε′′(f,T) for (**a**) neat PBSu and (**b**) PBSu_5% cutin. Highlighted are selected isothermal curves, whereas marked are the recorded relaxation processes (peaks).

**Figure 9:**
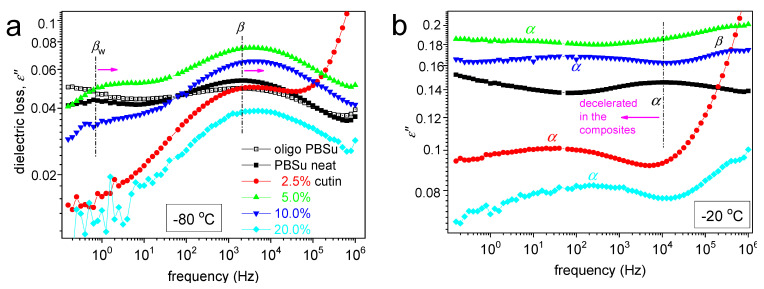
Selected DRS results in the form of comparative ε′′(f) for all systems at (**a**) −80 °C regarding local mobility and (**b**) −20 °C regarding segmental mobility.

Coming to molecular mobility, we present in Figure 8 and Figure 9 the results obtained by applying the advanced technique of dielectric spectroscopy [17]. Practically, we follow the various molecular motions by the relaxation of the corresponding dipole moments. This is achieved here by the recording of peaks of the imaginary part of dielectric permittivity, *ε*′′(*f*), related to the dielectric loss. At *T* < *T*_g_ any peaks (relaxations) are weak in magnitude and correspond to local dynamics, whereas when *T* approaches and exceeds *T*_g_, the dielectric signal enhances strongly, and the first strong peak recorded corresponds to segmental mobility. The latter is usually called as the main *α* relaxation and is considered to be the dielectric analogue of glass transition. At the higher temperatures of our recording, i.e., above 0 °C, a sharp signal rise is recorded and dominates the lower frequencies. This is due to various ionic conductivity phenomena, interfacial/electrode polarizations, etc. [17].

In Figure 8, we present two overall measurements of *ε*′′(*f,T*) at the examples of PBSu neat and PBSu/cutin 5%. Then, to provide a direct comparison between the different samples, we present in Figure 9 comparative isothermal spectra at fixed temperatures, one focusing on the local (Figure 8a) and another focusing on segmental dynamics (Figure 8b).

Regarding local mobility, two local relaxations are recorded in PBSu and dominate the signal also for all PBSu/cutin systems. These are the stronger and faster *β* and the weaker and slightly retardant *β*_w_ relaxations. Based on the few works in the literature [32,33], the *β* process originates from crankshaft rotations of the ester group (–C=O) in the backbone of the PBSu. On the other hand, *β*_w_ has been considered to exhibit similarities with other local processes originating from the relaxation of either hydrophilic sites with attached water molecules located at solid surfaces or hydrophilic polymer groups [34,35], and this has been found compatible in general in poly(*n*-alkylene succinates) [27], in particular, with its dielectric strength dependence from the *n*-alkylene sequence, as for increased *n* the fraction of the hydrophilic ester groups decreases. Therefore, it is considered that *β*_w_ monitors the dipolar mobility of the ester sites with attached water molecules and is possibly activated in the amorphous PBSu regions. 

Coming to the effects of cutin on PBSu, we may observe in Figure 9a, that *β* relaxation of PBSu is moderately accelerated (migrates to higher *f*) in the copolymers, suggesting enhanced mobility of the backbone ester groups. Most possibly, the effect is also connected with the expected lowering of the average polymer chain length due to the in situ polymerization of PBSu in the presence of cutin.

Regarding *β*_w_, the process is recorded within all samples except 2.5 and 20% cutin. Recalling the proposed origins of the process of the effect, may suggest for the latter samples, a severe fraction of –C=O of PBSu and cutin are engaged by other sites (e.g., –OH) and, thus, are not accessible to water molecules. From this point of view, this could be an additional indirect indication of the strong interaction between the two polymers; nevertheless, more work is needed to further clarify this point. 

Coming to the most interesting case of the segmental mobility (Figure 8b), the *α* process is significantly ‘decelerated’ (migrated toward lower *f*) in the copolymers as compared to neat PBSu. We recall that *α* relaxation is widely considered to screen the relaxation of dipole moments, being perpendicular to the polymer chain. Two phenomena would be expected here to dominate *α*, moreover, towards opposite directions. First, the PBSu-cutin interaction would introduce dynamical constraints and, thus, would impose a deceleration of *α*. Then, the drop of molar mass would be expected to accelerate the *α* process due to reduced chain–chain associations/entanglements and easier chains’ diffusion. The present recording of overall deceleration suggests the dominant character of the ‘grafting’ of a significant number of PBSu chains onto the cutin entities. 

We should note, from the methodological point of view, that the above effect is at the opposite direction compared to the DSC recordings, i.e., the mild drop in the calorimetric *T*_g_. Such and similar discrepancies between DSC and DRS have been presented in the past for complex polymeric systems. The discrepancy can be explained in terms of existence of ‘dynamical heterogeneities’ affecting in different ways the recordings of, in principle, different techniques [36,37].

### 3.5. Thermogravimetric Analysis (TGA)

**Figure 10:**
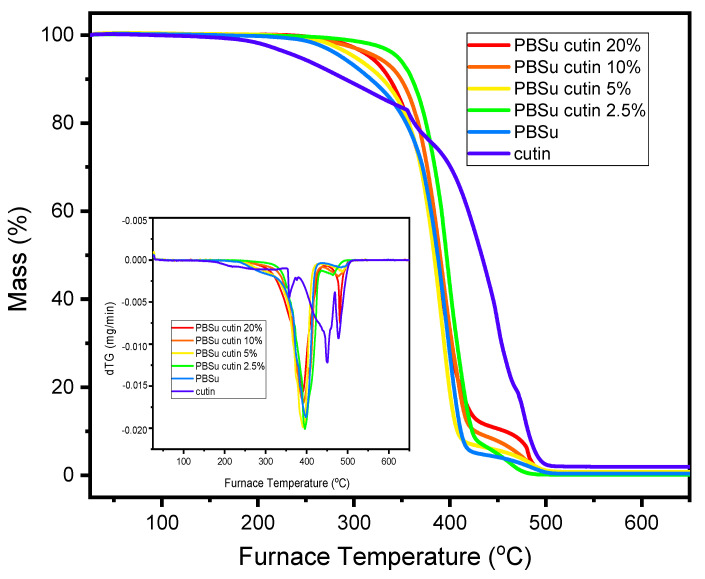
TGA-dTG analysis of the prepared copolyesters.

Thermogravimetric analysis results are shown in Figure 10. Neat cutin was thermally stable up to 200 °C. From this temperature, three mass loss steps were observed, which presented maximum mass loss rates at ca. 390, 470, and 550 °C. In the case of the prepared PBSu/cutin polyesters, the degradation begins at 300 °C (versus 250 °C for neat PBSu and 200 °C for neat cutin). The maximum mass loss for all PBSu/cutin prepared materials is approximately 420 °C. The degradation of copolymer occurs in two steps. The first until 400 °C is attributed mostly to PBSu decomposition, and the second that takes place between 400 and 490 °C, is attributed to decomposition of cutin. It is therefore concluded that PBSu/cutin copolyesters have improved thermal stability compared to neat PBSu and neat cutin, respectively. 

### 3.6. Water Contact Angle Measurements

**Figure 11:**
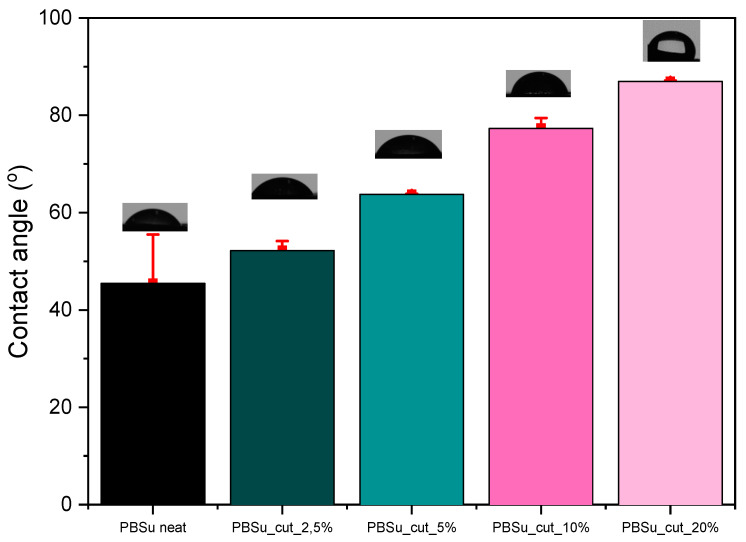
Contact angle measurements of the prepared materials.

The water contact angle (Figure 11) of polymeric materials depends on their chemical composition and surface properties, i.e., roughness, heterogeneity [38]. Due to its chemical composition and its biologic role, cutin is expected to enhance the hydrophobicity of the prepared materials. The result analysis of water contact angle measurements reveals that by increasing the percentage of cutin in the materials, the angle measured in the contact angle also increases, and therefore their hydrophobicity increases (Table 3). Among the materials measured, the most hydrophobic was found to be PBSu/cutin 20 wt%, which was expected given that cutin in nature is a polymeric lipid layer of plants that protects their cell wall by acting as a barrier to the entry of moisture and other components [39].

### 3.7. Polarized Light Microscopy (PLM)

**Figure 12:**
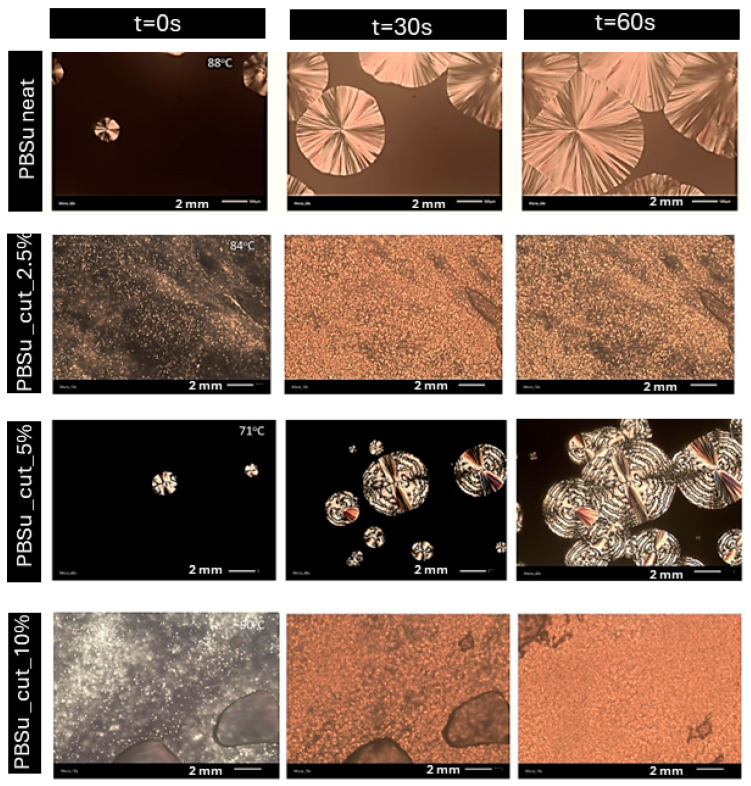
PLM photos of prepared PBSu/cutin polyesters.

The obtained PLM photos show that, in general, with the exception of the 5 wt% concentration, all samples exhibited a high nucleation density, indicating numerous small spherulites. The images obtained for the 2.5 and 10 wt% concentrations are indicative of heterogeneous crystallization, possibly suggesting the presence of impurities such as catalysts. The presence of cutin did not affect the size of the spherulites. However, the size of the crystals was notably larger for neat PBSu and PBSu/cutin 5 wt%, while significantly lower for concentrations of 2.5 and 10 wt% (Figure 12). 

The quantity of crystals formed exhibited an inverse relationship with their dimensions, for instance, the 2.5 and 10 wt% concentrations exhibited a significantly higher quantity of crystals compared to the 5 wt% concentration (Figure 12). In all copolymer cases, the rate of spherulitic growth was much higher compared to neat PBSu. Crystallization in the samples could be influenced by either molecular weight (lower molecular weight facilitating crystallization) or the presence of possible cross-linking bonds. Greater crosslinking led to more challenging crystallization. Comparing with DSC results, the crystallization temperature (*Tc*) increased with small amounts of cutin and gradually decreased with increasing concentration. Hence, crystallization was easier and faster at concentrations of 2.5, 5, and 10 wt%, whereas at 20 wt%, it was comparable to pure PBSu [40]. The effect could be compatible with the accelerated chains’ mobility (lower *T*_g_) in the copolymers, recorded in DSC. In general, the PLM results supplement the calorimetric observation on melt crystallization, discussed previously, whereas the recorded alternations in the semicrystalline morphology can be considered as an indirect tool to affect properties such as the mechanical performance, permeability, heat transport, etc. [41].

### 3.8. Soil Degradation and Enzymatic Hydrolysis

**Figure 13:**
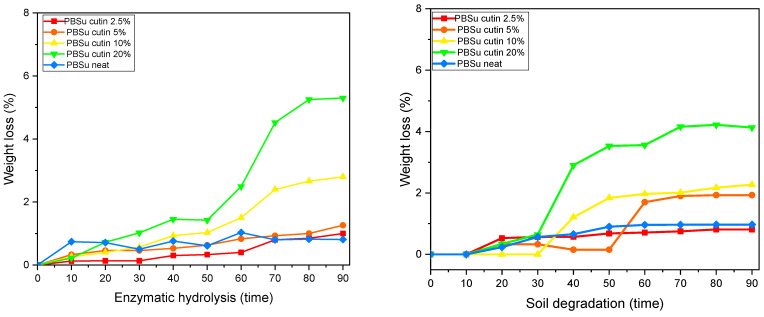
Weight loss during enzymatic hydrolysis (**left**), and soil degradation (**right**).

The biodegradation of polymers can be monitored with several different methods, including measuring their weight loss during incubation and soil burial in the presence of enzymes and microorganisms, and microscopic observation of their surfaces. To moderate the effect of dimensions and crystallinity, the specimens were films of the same size and similar thickness, which were kept at 37 °C. In Figure 13, the mass loss of PBSu copolymers for 90 days of (a) enzymatic hydrolysis and (b) soil degradation is reported [42].

PBSu is an aliphatic polyester, with a quite large number of methylene groups and β-ester bonds. As a result, enzymes like lipases can easily hydrolyze PBSu and its copolymers. In the present study, the enzymatic hydrolysis of prepared copolymers took place in an aqueous solution of Rhizopus Arrhizus and Pseudomonas Cepacia lipases at 37 °C. 

Weight loss of the samples expressed in percentage terms with the progress of time is presented in Figure 13 left As can be seen, neat PBSu lost less than 1% of its weight after 90 days of enzymatic hydrolysis. However, all composites had much higher weight losses and the rates were directly dependent on the cutin content of the prepared copolymers. It is worth noticing that PBSu/cutin at 20 wt% reached a high percentage of mass loss of almost 5.5% on day 90 [42].

The biodegradability of the prepared copolymers in soil was determined by measuring the mass loss of specimen buried in conventional leaf mold of the same size and similar thickness. Each specimen was buried in soil at room temperature (about 30 °C). The degree of soil degradation was estimated by the sample weight loss.

The humidity and enzymes present in soil can enter the copolymers, and compared with neat PBSu, the polymer degrades faster. Figure 13 right shows that PBSu/cutin at 10 wt% and 20 wt% lose more than 0.2% of their weight within the first month while neat PBSu hardly reaches 0.05% loss of weight within the second month. Cutin seems to facilitate the weight loss and degradation procedure of PBSu. 

### 3.9. Scanning Electron Microscopy (SEM)

**Figure 14:**
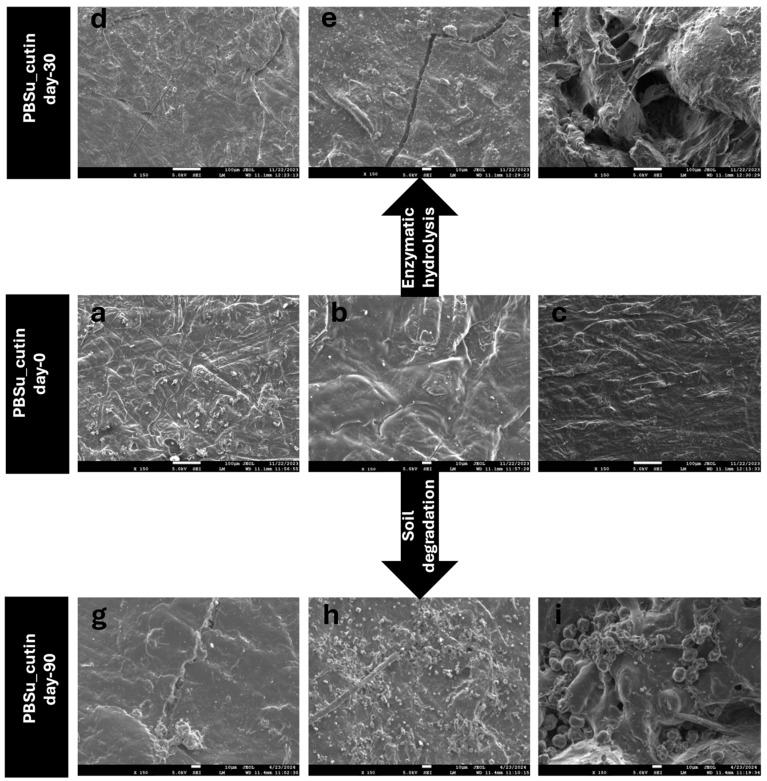
SEM images of PBSu/cutin copolymers before (**a**–**c**) and after (**d**–**f**) 30 days of enzymatic hydrolysis, and before (**a**–**c**) and after (**g**–**i**) 90 days of soil degradation.

The morphology of the sample’s surfaces upon 90 days of enzymatic hydrolysis and soil degradation was studied and captured with SEM (Figure 14). On day 0, cutin exhibits homogeneous distribution on PBSu/cutin-based copolymers and the surface of the prepared materials is even (Figure 14a, b, c, corresponds to PBSu/cutin 2.5, 5, 10% day 0, respectively). Cutin is known to be biodegradable and was expected to accelerate the decomposition of the materials. In fact, in photos g,h,i that correspond to PBSu/cutin 2.5, 5, 10% (day 90), respectively, we can observe that after 90 days of soil degradation, the cracks and holes of the surface of the materials appear deeper and bigger by increasing the proportion of cutin content. 

In a similar way, comparable behavior is also observed during the enzymatic hydrolysis of materials. In fact, in this case scenario, it is even more obvious that PBSu/cutin 2.5% (d) shows less cracks and an even surface compared to PBSu/cutin 5% (e), which starts to show small cacks on the surface and PBSu/cutin 10% (f), where the cracks are deeper and the surface is deformed. Obviously, the presence of PBSu provides permeation channels for water penetration as it decomposes, which facilitates the occurrence of hydrolytic degradation. These observations coincide with the mass loss measurements [43].

### 3.10. DSC Scans before and after Soil Degradation 

**Figure 15:**
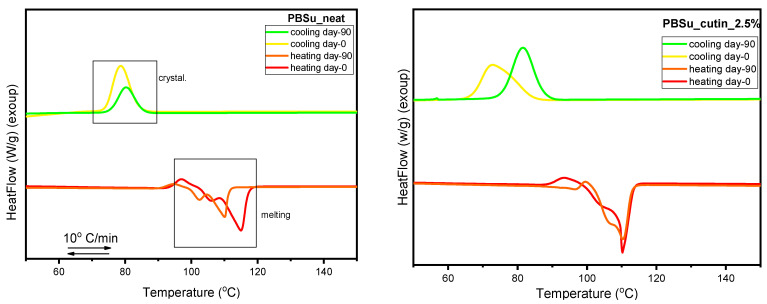

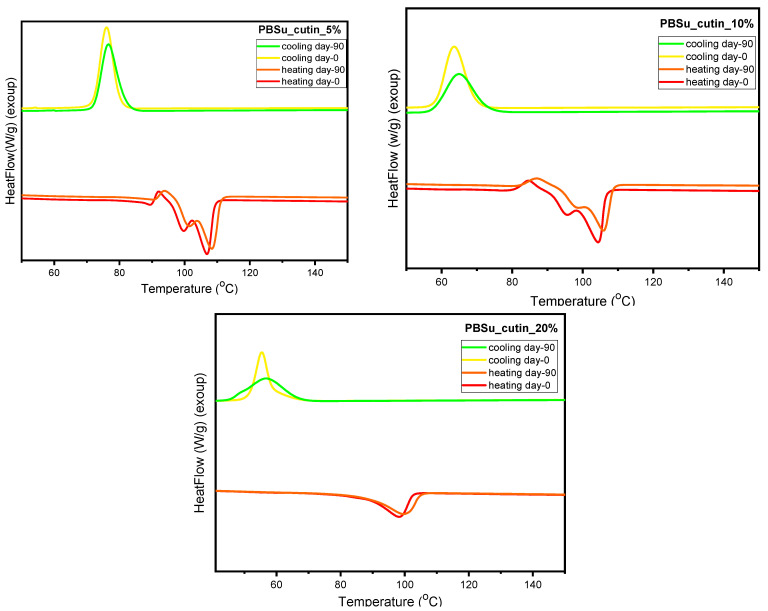
DSC scans of PBSu neat, PBSu cutin 2.5, 5, 10, 20 wt%.

Herein, the thermal behavior of the prepared PBSu/cutin polyesters prior and upon soil degradation (day 0 and day 90) is presented and compared in Figure 15. According to the literature, the amorphous regions in polyesters, such as PBSu, are usually more susceptible to microorganisms and enzymes than the crystalline regions. This is surely the case here, as at the soil conditions (*T* > *T*_g_ of PBSu), the amorphous fraction of the polymer is in the rubbery state; thus, permeation is facilitated. The biodegradation and the hydrolysis in the vulnerable aliphatic polyester units leads to a higher degree of structural regularity of the material during the degradation process. As a consequence, the crystalline fraction that is formed on cooling for PBSu samples after 90 days in soil is significantly reduced. Moreover, the formed crystals are slightly less stable than those formed in non-degraded PBSu (Figure 15 (top)) [44]. The addition of cutin characteristic groups to the polyester seems to have improved its thermal stability. More specifically, it is observed that with the addition of cutin, even after 90 days of soil degradation, the crystalline fraction and melting temperature of the PBSu/cutin remains practically constant and unchanged. Combing these effects with the previous data by FTIR on the increasing of the fraction of ‘bound ester groups’ in the copolymers (interactions between PBSu and cutin), we may conclude a ‘shielding’ effect of cutin on PBSu and, probably, vice versa. 

## 4. Conclusions

PBSu/cutin polyesters were successfully prepared via a two-stage melt polycondensation method and are reported in the present study for the first time. Their synthesis was extensively studied. Concerning the structural analysis of the prepared copolymers, the contribution of the cutin unit in the prepared polyesters was traced down to the peak corresponding to the ester group C=O, as observed in FTIR analysis. PBSu/cutin copolymers were semicrystalline according to XRD results and their main diffraction peaks were slightly shifted towards higher 2*θ* positions, as compared to neat PBSu, suggesting the formation of smaller unit cells. Melt crystallization was accelerated in the copolymers compared to neat materials, with the exception of PBSu/cutin 20 wt%, suggesting facilitated crystal nucleation, which was optimized for PBSu/cutin 2.5 wt%. The latter effect was also compatible with the lower *Tg* (enhanced chains’ mobility) in the copolymers. The aforementioned DSC results were supplemented by PLM. PLM showed that crystallization was easier and faster for PBSu/cutin 2.5, 5, and 10 wt%, whereas PBSu/cutin 20 wt% was comparable to pure PBSu. Concerning the effects of cutin on PBSu in terms of relaxation, it is accelerated in the copolymers, suggesting enhanced mobility of the backbone ester groups. The observed effect is also connected with the decrease in the average polymer chain length due to the in situ polymerization that indicates the dominant ‘grafting’ character of a significant number of PBSu chains onto the cutin entities. Due to its chemical composition and its biologic role, cutin enhanced the hydrophobicity of the prepared materials. Water contact angle measurements showed that increasing the ratio of cutin increases their hydrophobicity. Cutin is known to be biodegradable, and it was expected to accelerate the decomposition of the materials. The enzymatic hydrolysis and soil degradation studies, over a 90-days period, offered results that coincide with that. In fact, cutin seems to facilitate the weight loss and degradation procedure of PBSu, a result which is further supported by SEM images where the greater the cutin percentage of the copolymers, the bigger and deeper the cracks on their surface. Finally, DSC studies revealed that the crystalline fraction and the melting temperature of the PBSu/cutin remain practically constant and unchanged on day-90 of soil degradation. Combing these effects with the previous data by FTIR on the increasing of the fraction of ‘bound ester groups’ in the copolymers, we can conclude to a ‘shielding’ effect of cutin on PBSu. Overall, cutin seems to have a positive effect on PBSu properties, and the prepared polyesters are presented for potential biomedical applications such as drug delivery or tissue engineering. However, aiming to fully understand and optimize the copolymers for the aforementioned applications, more research and analyses, including antimicrobial testing and biocompatibility assessments, are required.

## Figures and Tables

**Table 1:** Ratios of used materials.

Name	Succinic Acid (g)	1,4 Butanediol (g)	Cutin (g)
PBSu/cutin_2.5%	40	25.5	1
PBSu/cutin_5%	40	25.5	2
PBSu/cutin_10%	40	25.5	4
PBSu/cutin_20%	40	25.5	8

**Table 2:** The samples under investigation and characteristic values obtained by DSC. Melt/hot crystallization temperature and crystalline fraction, *T*_c1,2_ and CF_c_, respectively, glass transition temperature, *T*_g_, and heat capacity change, Δ*c*_p_, cold crystallization.

Sample	*T*_c1_ (°C)	*T*_c2_ (°C)	CF_c_ (wt%)	*T*_g_ (°C)	Δ*c*_p_ (J/g∙K)	*T*_cc_ (°C)	CF_cc_ (wt%)	*T*_m,max_ (°C)	CF_m_ (wt%)
PBSu neat	64	-	38	−33	0.23	84	6	108	40
PBSu/cutin 2.5%	82	49	37	−34	0.21	-	-	113	37
PBSu/cutin 5%	67	49	39	−37	0.21	90	4	110	40
PBSu/cutin 10%	74	47	38	−35	0.23	-	-	108	38
PBSu/cutin 20%	46	-	30	−35	0.22	-	-	100	33

**Table 3:** Contact angle measurements.

Name	Angle 1	Angle 1	Angle 2	Angle2	Angle Average
	t = 0	t = 30	t = 0	t = 30	
PBSu neat	43.58	38.37	53.44	52.56	45.465
PBSu/cutin 2.5 wt%	54.95	54.81	54.76	53.59	54.2
PBSu/cutin 5 wt%	68.06	58.82	65.24	63.65	61.235
PBSu/cutin 10 wt%	81.5	78.84	77.19	75.82	77.33
PBSu/cutin 20 wt%	87.8	87.1	87.1	86.8	86.95

## Data Availability

The original contributions presented in the study are included in the article, further inquiries can be directed to the corresponding authors.
